# In Vitro Evaluation of Ferutinin Rich-*Ferula communis* L., ssp. *glauca*, Root Extract on Doxorubicin-Induced Cardiotoxicity: Antioxidant Properties and Cell Cycle Modulation

**DOI:** 10.3390/ijms241612735

**Published:** 2023-08-13

**Authors:** Roberta Macrì, Irene Bava, Federica Scarano, Rocco Mollace, Vincenzo Musolino, Micaela Gliozzi, Marta Greco, Daniela Foti, Luigi Tucci, Jessica Maiuolo, Cristina Carresi, Annamaria Tavernese, Ernesto Palma, Carolina Muscoli, Vincenzo Mollace

**Affiliations:** 1 Pharmacology Laboratory, Institute of Research for Food Safety and Health IRC-FSH, Department of Health Sciences, University Magna Graecia of Catanzaro, 88100 Catanzaro, Italy; robertamacri85@gmail.com (R.M.); irenebava@libero.it (I.B.); micaela.gliozzi@gmail.com (M.G.); l.tucci@head-sa.com (L.T.); an.tavernese@gmail.com (A.T.); muscoli@unicz.it (C.M.); mollace@libero.it (V.M.); 2 Department of Cardiology, IRCCS San Raffaele Pisana, 00166 Rome, Italy; 3 Division of Cardiology, Fondazione Policlinico Tor Vergata, 00133 Rome, Italy; 4 Pharmaceutical Biology Laboratory, Institute of Research for Food Safety and Health IRC-FSH, Department of Health Sciences, University Magna Graecia of Catanzaro, 88100 Catanzaro, Italy; v.musolino@unicz.it (V.M.); maiuolo@unicz.it (J.M.); 5 Department of Health Sciences, University Magna Graecia of Catanzaro, 88100 Catanzaro, Italy; marta.greco@unicz.it (M.G.); foti@unicz.it (D.F.); 6 Veterinary Pharmacology Laboratory, Institute of Research for Food Safety and Health IRC-FSH, Department of Health Sciences, University Magna Graecia of Catanzaro, 88100 Catanzaro, Italy; carresi@unicz.it (C.C.); palma@unicz.it (E.P.); 7 Renato Dulbecco Institute, Lamezia Terme, 88046 Catanzaro, Italy

**Keywords:** *Ferula* L., Ferutinin, Doxorubicin, cardiotoxicity, cardioprotection, ROS, cell cycle modulation

## Abstract

The clinical use of anthracycline Doxorubicin as an antineoplastic drug in cancer therapy is limited by cardiotoxic effects that can lead to congestive heart failure. Recent studies have shown several promising activities of different species of the genus *Ferula* belonging to the *Apiaceae Family*. *Ferula communis* is the main source of Ferutinin—a bioactive compound isolated from many species of *Ferula*—studied both in vitro and in vivo because of their different effects, such as estrogenic, antioxidant, anti-inflammatory, and also antiproliferative and cytotoxic activity, performed in a dose-dependent and cell-dependent way. However, the potential protective role of Ferutinin in myocardium impairment, caused by chemotherapeutic drugs, still represents an unexplored field. The aim of this study was to test the effects of Ferutinin rich-*Ferula communis* L. root extract (FcFE) at different concentrations on H9C2 cells. Moreover, we evaluated its antioxidant properties in cardiomyocytes in order to explore new potential therapeutic activities never examined before in other experimental works. FcFE, at a concentration of 0.25 µM, in the H9C2 line, significantly reduced the ROS production induced by H_2_O_2_ (50 µM and 250 µM) and traced the cell mortality of the H9C2 co-treated with Ferutinin 0.25 µM and Doxorubicin (0.5 µM and 1 µM) to control levels. These results showed that FcFE could protect against Doxorubicin-induced cardiotoxicity. Further molecular characterization of this natural compound may open the way for testing FcFE at low concentrations in vivo and in clinical studies as an adjuvant in cancer therapy in association with anthracyclines to prevent side effects on heart cells.

## 1. Introduction

Cancer and cardiovascular diseases (CVD) are the leading causes of death worldwide [1,2].

Although cancer and cardiovascular diseases are considered two distinct entities, they have common clinical characteristics and potential interactions, such as chronic inflammation—an essential hallmark of the pathogenesis and progression of both diseases [3,4]. Despite new cancer drugs having improved the life expectancy of cancer patients, the acute and long-term side effects of cancer chemotherapy remain an unsolved problem in the management of oncological disease [5,6].

Indeed, anticancer therapy is often associated with an increased risk of developing cardiovascular toxicity that could affect ongoing cancer treatment [7].

Among the chemotherapeutic agents capable of inducing severe cardiovascular side effects, anthracyclines are a well-known example [8].

Doxorubicin (Doxo) is an anthracycline derived from *Streptomyces peucetius* bacterium and commonly used in the treatment of solid tumors in adult and pediatric patients [9]. Doxo cytotoxic activity is not restricted to cancerous cells: its amphoteric property allows Doxo to translocate into a variety of subcellular compartments, disrupting their integrity [10]. In particular, Doxo leads to mitochondrial dysfunction, Reactive Oxygen Species (ROS) production, lipid peroxidation and DNA damage, impaired calcium handling, and induction of p53 and apoptotic pathways. Furthermore, Doxo blocks DNA synthesis through suppression of DNA Topoisomerase II Beta (TOP2b) and the intercalation into DNA bases [11,12,13,14].

Since Doxo cannot differentiate between normal and cancer cells, the damage to cardiac cells is a result of its action on noncancerous cells; in particular, its long-term use is limited by cumulative dose-related cardiotoxic effects that can lead to irreversible cardiomyopathy and congestive heart failure [15].

Several pieces of evidence have shown that both mitochondrial-dependent [16] and independent [17] pathways are involved in Doxo-induced cardiac cell death and that the transcription factor p53 is upstream of the events that lead to mitochondrial activation of the apoptotic pathway [18]. Upon administration, Doxo binds cardiolipin and accumulates in the inner mitochondrial membrane (IMM), where it creates a redox cycle resulting in ROS production [19]. Uncontrolled ROS production induces mitochondrial DNA damage, leading to activation of downstream proapoptotic pathways and impaired myocardial contractile performance [20]. ROS are produced by mitochondrial oxidative metabolism as well as response to inflammation and bacterial invasion. Oxidative stress refers to the imbalance between the excessive ROS or oxidants production and the cell capability to trigger an effective antioxidant response. Intracellular ROS accumulation and the resulting damage are involved in several pathological conditions including diabetes, atherosclerosis, neurodegeneration, cancer, and aging [21].

Recent studies highlighted that nutraceutical supplementation in cancer therapy may exert synergistic effects by inhibiting cancer cells proliferation and differentiation and reducing chemotherapy side effects [22,23]. In particular, flavonoids and sesquiterpenes may provide cardiovascular protection against Doxo-induced toxicity thanks to their antioxidant and anti-inflammatory effects [24,25,26].

Among these, 7-Monohydroxyethylrutoside (monoHER) is a flavonoid belonging to the semisynthetic hydroxyethylrutoside family that has been shown to inhibit negative cardiac effects in a dose-dependent manner both in preclinical [27] and clinical trials [28].

Its anti-inflammatory action is related to the reduction of N- e-[carboxymethyl]-lysine, the accumulation of which is promoted by Doxo during cardiotoxicity [28]. This dose-dependent monoHER cardioprotective effect does not affect the antitumor capacity of Doxo [29].

Other flavonoids, such as catechins, have cardioprotective properties at low doses through the chelating activity of iron [30]. Quercetin, in addition to its high antioxidant capacity, can inhibit TOP2 and intercalate into DNA strands, thereby boosting the antitumor effect of Doxorubicin [31,32]. Resveratrol, thanks to its anticancer, anti-inflammatory and antioxidant properties, was able to prevent Doxo-induced toxicity in cardiac H9c2 cells through inhibition of intracellular ROS accumulation [33,34,35].

Furthermore, Carresi et al. observed that Bergamot Polyphenolic Fraction (BPF), thanks to its ability to counteract ROS production and restore protective autophagy, reverted the cardiotoxic effects of Doxo, associated with enhanced cardiac stem cell (CSC) survival [36].

Among the plants richest in sesquiterpenes and their derivatives, the genus *Ferula* L. is one of the most representative. *F. communis* L. was used in traditional medicine to counteract hysteria and dysentery. The rhizomes of this plant were locally used as a remedy for the treatment of skin infections, while the roasted flower buds were used against fever [37].

The uses of non-poisonous *F. communis* L. as a phytohormone were attributed to Ferutinin [38], a daucane ester of a sesquiterpenic alcohol, isolated for the first time from *Ferula tenuisecta* Eug. Kor. This plant acts as a possible source of phytoestrogens. In Morocco, *F. communis* L. has traditionally been used as a hypoglycemic medicinal plant [39] but its use has been restricted due to its toxicity [37].

Ferutinin, obtained from the plants *Ferula ovina* Boiss., *Ferula hermonis* Boiss., *Ferula communis* L., and other species, has shown estrogenic, antioxidant, anti-inflammatory, antiproliferative, and cytotoxic activities in a dose-dependent way [40,41,42,43,44]. It has been established that Ferutinin has protective potential at low doses and severe toxic effects at high doses. Indeed, low concentrations of Ferutinin are associated with an important antioxidant action; conversely, in a dose-dependent manner, high concentrations of Ferutinin are able to increase the permeability of mitochondria, thymocytes, liposomes, sarcoplasmic reticulum, and bilayer lipid membranes for cations, especially Ca^2+^ [41].

The structural similarity with steroid hormones classified Ferutinin as a phytoestrogen, with a significant affinity for both estrogen receptors, alpha (ERα) and beta (ERβ), and for the G-protein-coupled estrogen receptor (GPER) [45].

An in vitro study showed that *Ferula communis* L. root extract, thanks to its estrogenic-like properties, may increase the efficacy of tamoxifen for the treatment of hormone-receptor-positive breast cancer [46]. In particular, the additive effect of *Ferula* L. root extract allows for the use of lower concentrations of the chemotherapy drug, reducing tamoxifen side effects [46]. The ability of Ferutinin to induce cytotoxicity, at defined concentrations, appeared to be directed in particular to cancer cells. In fact, a comparative study, involving two cancer lines (MCF-7 and Hela) and a normal one (HBL-100), showed a lower Ferutinin toxicity in healthy cells with respect to cancer cells [46]. This result reinforces the concept that Ferutinin activity could be dose- and cell type dependent.

On the basis of these evidence, and considering the deleterious cardiac effect of anthracyclines—particularly that of Doxo—we tested the potential protective effect of *Ferula communis* L. root extract concentrated in Ferutinin (FcFE) to counteract the Doxo-induced cardiotoxic effect. The choice to test the cardiac beneficial effect of FcFE on rat H9c2 embryonic cardiomyocytes is due to its common use in in vitro studies, including cardiotoxicity analyses of drugs, such as anthracycline toxicity. Indeed, previous results highlighted the cardiotoxic effect of Doxo on H9c2 and the potential protective effects of natural derivatives [24,25]. Furthermore, in vitro evidence demonstrated that H9c2 cells are more similar to primary cardiomyocytes than mouse atrial HL-1 cells with regard to energy metabolism patterns and oxidative stress modulation [47].

## 2. Results

### 2.1. Effects of FcFE on H9c2 Viability

**Figure 1:**
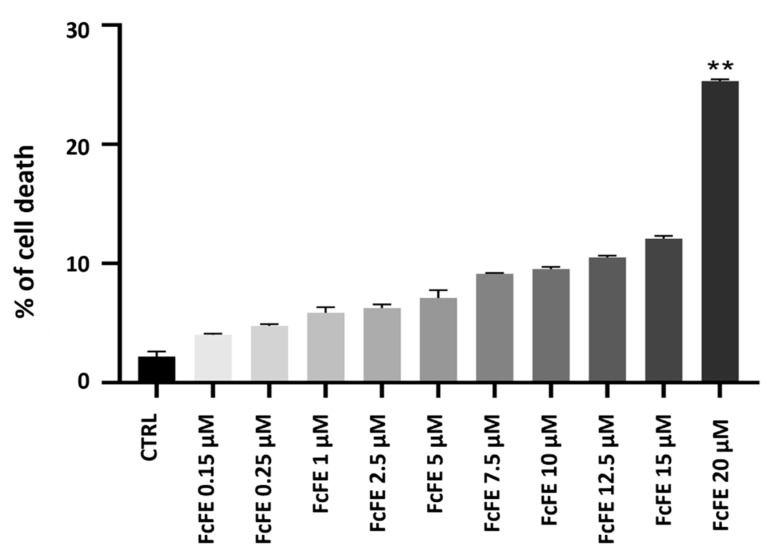
Evaluation of cell viability. Cell viability was measured after treatment with FcFE for 24 h. The figure shows the percentage of cell death induced by FcFE at increasing concentrations. The values of three independent experiments performed in triplicate are expressed as mean ± S.E.M. **: *p* < 0.01 vs. CTRL.

Incubation of H9c2 cells (for 24 h, into a humidified 5% CO_2_ atmosphere at 37 °C) with different concentrations of FcFE, corresponding to 0.15 μM, 0.25 μM, 1 μM, 2.5 μM, 5 μM, 7.5 μM, 10 μM, 12.5 μM, 15 μM, and 20 μM of Ferutinin, did not induce significant mortality compared with the CTRL group. A significant mortality was observed only when cells were incubated with FcFE 20 μM (Figure 1). DMSO, present in FcFE dilutions (used for treatment) at low concentrations (≤0.08%), did not have an effect on cell viability. 

### 2.2. Effect of FcFE on Cell Metabolic Activity

**Figure 2:**
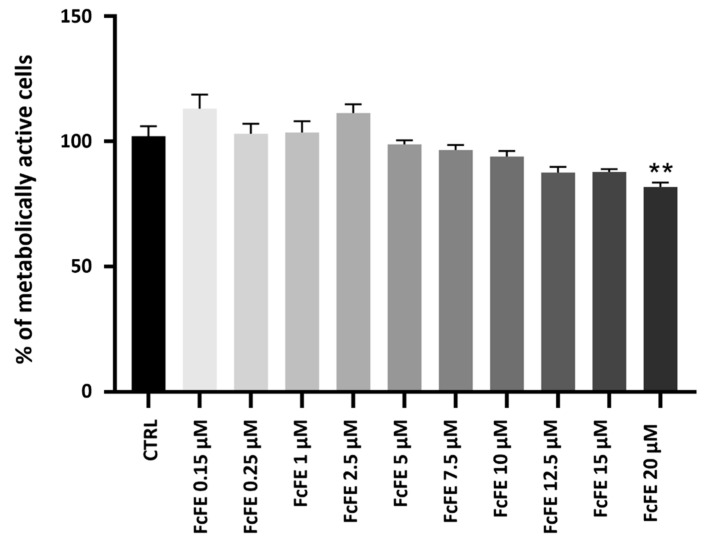
Evaluation of cell metabolic activity. Cell metabolic activity was assessed after treatment with FcFE for 24 h. The figure shows the percentage of metabolic activity on H9c2 cells treated with FcFE at increasing concentrations. The values of three independent experiments performed in triplicate are expressed as mean ± S.E.M. **: *p* < 0.01 vs. CTRL.

Incubation of H9c2 cells (for 24 h, into a humidified 5% CO_2_ atmosphere at 37 °C) with different concentrations of FcFE, corresponding to 0.15 μM, 0.25 μM, 1 μM, 2.5 μM, 5 μM, 7.5 μM, 10 μM, 12.5 μM, 15 μM, and 20 μM of Ferutinin, did not induce a significant reduction in cell metabolic activity compared with the CTRL group. A significant reduction in metabolic activity was observed in cells incubated with FcFE 20 μM (Figure 2). DMSO, present in FcFE dilutions (used for treatment) at low concentrations (≤0.08%), did not have an effect on cell metabolic activity.

### 2.3. Effect of FcFE on H_2_O_2_-Induced ROS

**Figure 3:**
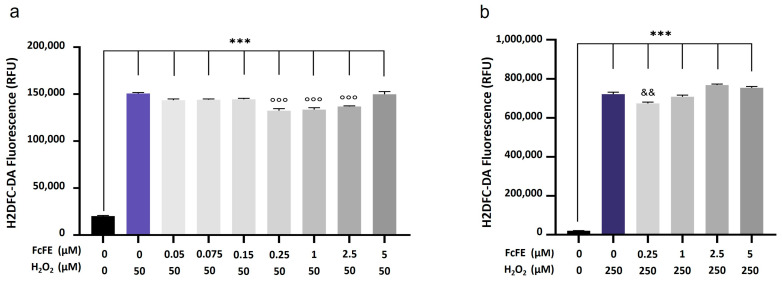
Evaluation of ROS production. In cultured H9c2 cells incubated with H_2_O_2_ 50 μM (**a**), pre-treatment with FcFE 0.25 μM, 1 μM, and 2.5 μM led to a significant reduction in ROS production (assessed by H2DCF-DA fluorescence determination after 30 min of incubation and indicated as RFU). In cells incubated with H_2_O_2_ 250 μM (**b**), pre-treatment with 0.25 μM of FcFE induced a significant reduction in free radical release. The values of three independent experiments performed in triplicate are expressed as mean ± S.E.M. ***: *p* < 0.001 vs. CTRL; °°°: *p* < 0.001 vs. H_2_O_2_ 50 μM; &&: *p* < 0.01 vs. H_2_O_2_ 250 μM.

Cultured H9c2 cells incubated (for 30 min, into a humidified 5% CO_2_ atmosphere at 37 °C) with H_2_O_2_ 50 μM or 250 μM induced a significant intracellular production of ROS. In cells incubated with H_2_O_2_ 50μM, a pre-treatment with 0.05 μM, 0.075 μM, and 0.15 μM of FcFE, showed a reduction in ROS production (assessed by H2DCF-DA fluorescence determination), which did not reach statistical significance. A pre-treatment with higher concentrations of FcFE (0.25 μM, 1 μM, and 2.5 μM) led to a significant attenuation of H_2_O_2_-induced free radical release (Figure 3a). A concentration of FcFE 5 μM had no effect on the intracellular production of ROS (Figure 3a). In cells incubated with H_2_O_2_ 250 μM, a pre-treatment with FcFE 0.25 μM induced a significant reduction in free radical release, whereas higher concentrations of FcFE had no effect on ROS production (Figure 3b).

### 2.4. Effect of FcFE Pre-Treatment on Doxo-Induced ROS

**Figure 4:**
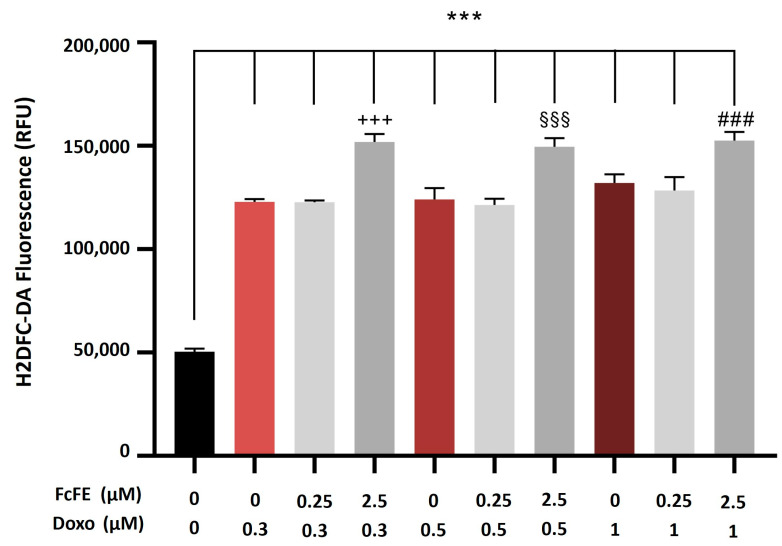
Evaluation of ROS production. H9c2 incubated with Doxo showed a significant concentration-dependent intracellular production of ROS (assessed by H2DCF-DA fluorescence determination and indicated as RFU). FcFE 0.250 μM showed no significant reduction in ROS production in cells incubated with DOXO 1 μM. In H9c2 cells incubated with DOXO at increasing concentrations, pre-treatment with FcFE 2.5 μM exerted significant additional ROS accumulation activity. Results were confirmed by three independent experiments performed in triplicate. The values of three independent experiments are expressed as mean ± S.E.M. ***: *p* < 0.001 vs. CTRL; +++: *p* < 0.001 vs. Doxo 0.3 μM; §§§: *p* < 0.001 vs. Doxo 0.5 μM; ###: *p* < 0.001 vs. Doxo 1 μM.

H9c2 cells incubated (for 24 h, into a humidified 5% CO_2_ atmosphere at 37 °C) with increasing concentrations of DOXO (Doxo) (0.3 μM, 0.5 μM, and 1 μM) showed a significant concentration-dependent intracellular production of ROS (Figure 4). In cells incubated with DOXO 1 μM, a pre-treatment with FcFE 0.25 μM showed a reduction trend in ROS production that was not significant, whereas a pre-treatment with FcFE 2.5 μM exacerbated the effect of Doxo-induced free radical release (Figure 4).

### 2.5. Effect of FcFE Pre-Treatment on Doxo-Induced Cell Death

**Figure 5:**
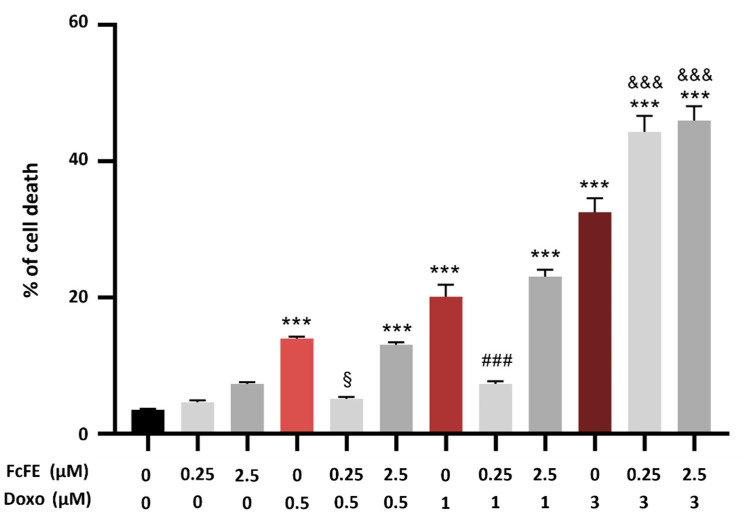
Evaluation of cell viability. In H9c2 cells exposed to Doxo concentrations of 0.5 μM and 1 μM, FcFE 0.25 μM significantly reduced cell death in cells. In H9c2 cells exposed to Doxo concentration of 3 μM, FcFE concentrations of 0.25 μM and 2.5 μM significantly exacerbated cell death. The values of three independent experiments performed in triplicate are expressed as mean ± S.E.M. ***: *p* < 0.001 vs. CTRL; §: *p* < 0.5 vs. Doxo 0.5 μM; ###: *p* < 0.001 vs. Doxo 1 μM; &&&: *p* < 0.001 vs. Doxo 3 μM.

For evaluation of FcFE pre-treatment on Doxo-induced cytotoxicity, H9c2 cells were pre-treated for 3 h with FcFE (0.25 μM–2.5 μM); then, the cells were treated with increasing concentrations of Doxo (0.5 μM, 1 μM, and 3 μM) for 24 h (into a humidified 5% CO_2_ atmosphere at 37 °C). As shown in Figure 5, Doxo-induced cytotoxicity at any concentration was tested. FcFE 0.25 μM significantly reduced cell death in cells incubated with Doxo 0.5 μM or Doxo 1 μM (Figure 5). FcFE 2.5 μM exacerbated the extent of cell death induced by DOXO 0.5 μM and 1 μM (Figure 5). Instead, pre-treatment with FcFE 0.25 μM and 2.5 μM exacerbated the effect of DOXO 3 μM treatment (Figure 5).

### 2.6. Effect of FcFE Pre-Treatment on Doxo-Induced Metabolic Activity Inhibition

**Figure 6:**
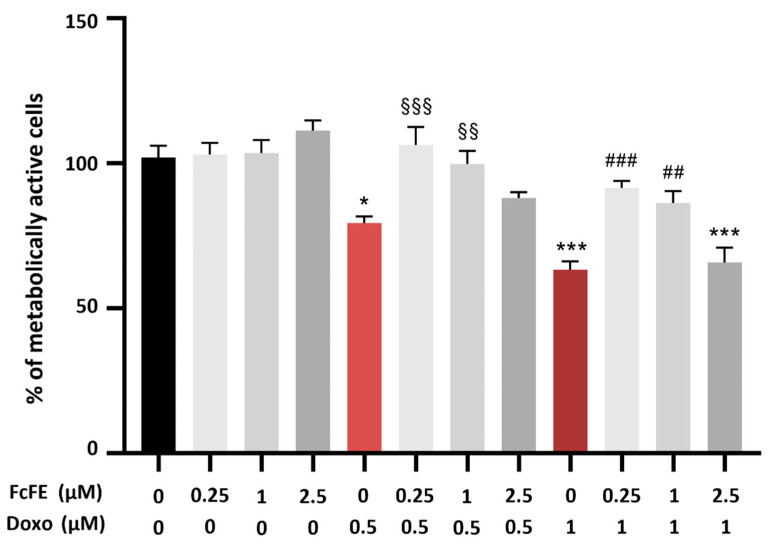
Metabolic activity analysis. The figure shows the effects of Doxo 0.5 μM and 1 μM on cell metabolic activity. H9c2 cells were treated with the indicated concentrations of FcFE for 24 h and then analyzed by MTT assay. The pre-treatment with FcFE at all concentration restored metabolic activity in H9c2 cells exposed to Doxo concentrations of 0.5 μM and 1 μM. FcFE 2.5 μM significantly exacerbated cell death in cells treated with Doxo 1 μM. CTRL, cells without treatment. The graph is representative of three independent experiments performed in triplicate. The values are expressed as mean ± S.E.M. ***: *p* < 0.001 vs. CTRL; * *p* < 0.05 vs. CTRL; §§§: *p* < 0.001 vs. Doxo 0.5 μM; §§: *p* < 0.01 vs. Doxo 0.5 μM; ###: *p* < 0.001 vs. Doxo 1 μM; ##: *p* < 0.01 vs. Doxo 1 μM.

Doxo-induced modifications in cellular metabolic activity and the potential protective role of FcFE pre-treatment were evaluated by MTT assay. H9c2 cells were pre-treated for 3 h with FcFE (0.25 μM–2.5 μM); then, the cells were treated with increasing concentrations of Doxo (0.5 μM and 1 μM) for 24 h (into a humidified 5% CO_2_ atmosphere at 37 °C). As shown in Figure 6, Doxo induced a significant reduction in cell metabolic activity, which is prevented through the pre-treatment with FcFE 0.25 μM and 1 μM; moreover, pre-treatment with FcFE 2.5 μM exacerbated the effect of Doxo 3 μM treatment (Figure 6).

### 2.7. FcFE Restores Cell Cycle in H9c2 Myoblasts Incubated with Doxo

**Figure 7:**
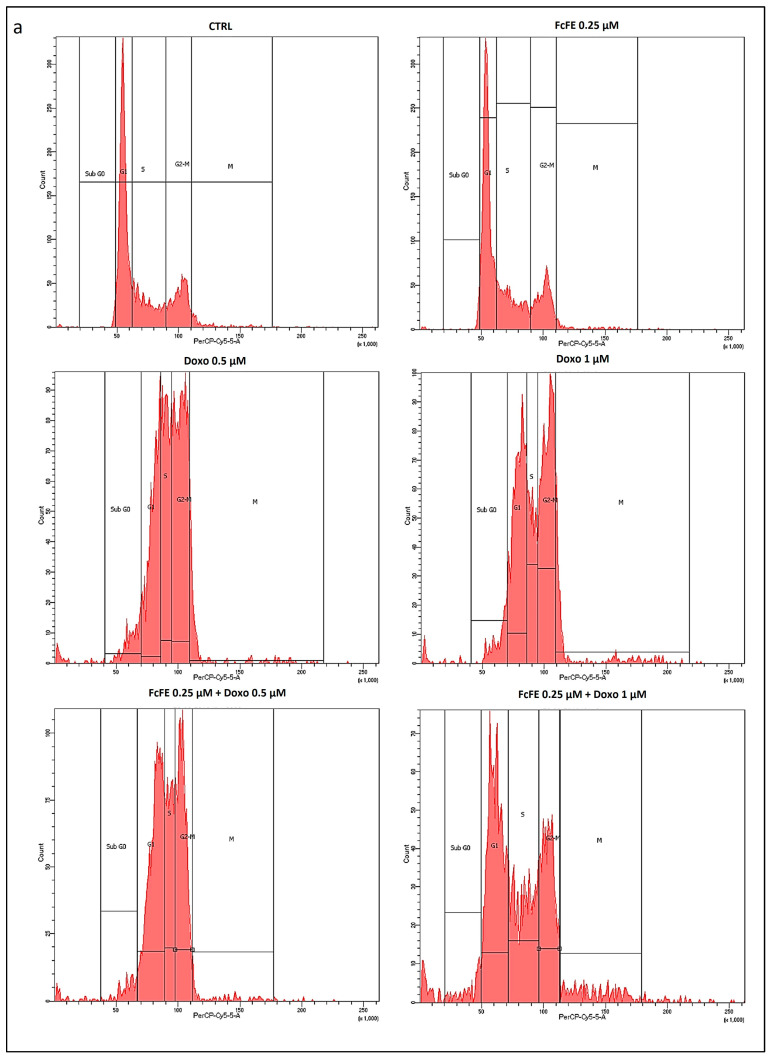

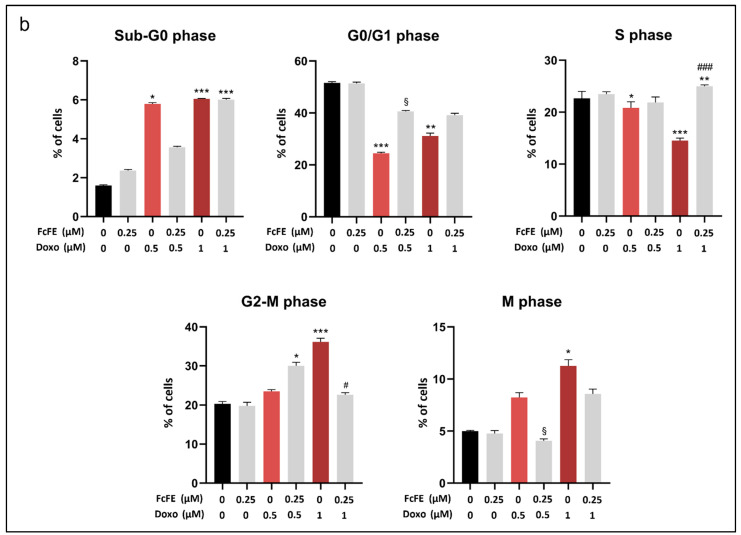
Cell cycle analysis. The panels and the histograms ((**a**) and (**b**), respectively) show the effects of FcFE 0.25 μM, Doxo 0.5 μM, and Doxo 1 μM on cell cycle phases and the activity exerted by the pre-treatment with FcFE 0.25 μM on cell cycle progression in H9c2 exposed to Doxo 0.5 μM and Doxo 1 μM, respectively. Cell lines were treated with the indicated concentrations of the compounds for 24 h and then analyzed by FACS, as indicated in methods section. CTRL, cells without treatment. The graphs are representative of three independent experiments performed in triplicate. The values are expressed as mean ± S.E.M. ###: *p* < 0.001; #: *p* < 0.05 vs. CTRL. ***: *p* < 0.001; **: *p* < 0.01 vs. Doxo 0.5 μM; * *p* < 0.05 vs. CTRL; §: *p* < 0.5 vs. Doxo 0.5 μM.

No differences in cell cycle progression were observed in H9c2 cells exposed to FcFE 0.25 μM with respect to untreated cells (CTRL) (Figure 7a,b). The S phase reduction induced by Doxo 0.5 μM and Doxo 1 μM treatment showed significant dose-dependence (Figure 7a,b). Flow cytometry analysis showed that H9c2 cells treated with Doxo 1 μM were highly accumulated in the G2-M phase with a concomitant decrease in the number of cells in the G0-G1 and S phases of the cell cycle (Figure 7a,b). In the cells incubated with Doxo 1 μM, pre-treatment with FcFE 0.25 μM led to a significant recovery of G2-M and the S phase of the cell cycle (Figure 7a,b).

## 3. Discussion

Cardiotoxicity is one of the most frequent clinical complications observed during anticancer chemotherapy; it is particularly dangerous during anthracycline therapy. Among these, Doxo clinical application is limited due to dose-dependent congestive cardiac failure development [48,49].

A very promising strategy to reduce Doxo-cardiotoxicity consists in the use of cardioprotective agents or preparations derived from natural products. Thanks to their low toxicity and high antioxidant activity, they may represent a potential therapeutic approach to protect cardiac muscle from the detrimental effects of oxidative stress [27,49,50,51].

In addition, the combination of natural bioactive compounds with traditional chemotherapeutic drugs can enhance anticancer efficacy [52].

The results obtained in different experimental works have shown that natural derivatives, such as flavonoids and sesquiterpenes, were able to reduce Doxo-toxicity owing to their antioxidant, anti-inflammatory, and antitumor properties [53,54].

Because of these properties, the use of flavonoids and sesquiterpenes as possible therapeutic agents to protect from Doxo-induced toxicity has become a focus of current research [27,51].

Among them, Ferutinin—a bioactive compound extracted from many species of plants of the genus *Ferula* and already known for its anticancer, antimicrobial, antiviral, and anti-inflammatory properties—is recognized for its protective and antioxidant effects exerted at low doses [55].

Since current treatments used to improve the prognosis of patients with Doxo-induced cardiomyopathy are not effective, in our in vitro study we evaluated the concentration-dependent effects of Ferutinin contained in *Ferula communis*, ssp. *glauca*, powder (FcFE) on the H9c2 rat cardiomyoblast cell line. The chemical composition of our powder determined by HPLC revealed a Ferutinin concentration of 27%, with Jaeskeanadiol Benzoate and Teferin, respectively, at 1.7 and 0.7%, suggesting that the effect of the extract following the serial dilutions carried out for cells treatment is mainly due to Ferutinin activity. Our results obtained after the treatment of H9c2 cells with different increasing concentrations of FcFE showed that low concentrations of FcFE did not produce significant changes in viability. Significant mortality was observed only when cells were incubated with FcFE at a concentration of 20 μM.

Indeed, several evidence showed that sesquiterpenes have a protective potential at low doses and an aggravating toxicity effect at high doses [56].

In particular, low doses of the sesquiterpene Ferutinin have a high antioxidant, anti-inflammatory, and antitumor activity thanks to a reduction in the cellular oxidative status and due to the ability of the sesquiterpenes to oxidize over time into sesquiterpenols [41]. An in vivo study demonstrated that Ferutinin has a neuromodulator activity, with anticonvulsant properties at low doses, suggesting it to be a potential scavenger fighting against the range of neurodegenerative diseases by improving the total antioxidant potential [55]. Indeed, Ferutinin in an in vivo study was able to modulate glycine receptors, a key mediator of synaptic signaling in central nervous system regions. In this study, mice treated with Ferutinin at low doses were protected from the toxic effects of strychnine. Nevertheless, the highest dose of Ferutinin aggravated strychnine toxicity. This biphasic effect of Ferutinin, protective at low doses and aggravating toxicity at high doses, towards glycine receptors was observed previously in elevation of intracellular calcium in leukemia human Jurkat T-cell line and Zn^2+^ ions on the strychnine-sensitive glycine receptor [55].

These results are corroborated by evidence whereby Ferutinin significantly increases the antioxidant enzymes, such as catalase, superoxide dismutase, and glutathione peroxidase, and reduces liver lipid peroxidation [43]. Therefore, starting from the evidence of antioxidant power of Ferutinin at low concentrations, we wanted to assess the antioxidant effect of low concentrations of FcFE in the presence of H_2_O_2,_ at two different increasing concentrations. Exposure to H_2_O_2_ is a widely used method to cause oxidative damage/stress in cell models [57]. The most commonly used concentrations of H_2_O_2_ to generate oxidative damage in cellular models are in the high micromolar range; notably, the 100–500 µM range was used in half of the publications [57]. Previous studies in different cell lines showed that the treatment time, with H_2_O_2_ used to produce an accumulation of ROS (30 min), did not cause a reduction in cell viability. In order to obtain a significant reduction in viability, it is necessary to treat cells for 3 h or more. Thus, exposure to H_2_O_2_ for 30 min causes only oxidative damage in treated cells [58]. According to previous data, the cytotoxicity of H_2_O_2_ treatment at a concentration of 250 µM in the H9c2 cell line, including caspase activation, was observed only after 2 h of treatment [59]. Our results showed a significant reduction in ROS production at low concentrations of FcFE—specifically, 0.25 μM, 1 μM, and 2.5 μM—in cells treated with a lower concentration of H_2_O_2_ (50 μM) and only 0.25 μM in cells treated with a higher concentration of H_2_O_2_ (250 μM). The results obtained showed that in the presence of a higher oxidant stimulus, the FcFE at high concentrations produced an additive effect on oxidative status, losing its antioxidant potential.

Since several reports have already shown that Doxo and other members of the anthracycline family are able to induce ROS production and to be redox cycled [60,61], subsequently, we have evaluated the potential protective effect of low concentrations of FcFE on Doxo-induced ROS production. The cells pre-treated with 0.25 μM of FcFE and exposed to increasing concentrations of Doxo showed only a non-significant trend of reduction in ROS levels. Moreover, 2.5 μM of FcFE produced a significant additive effect on Doxo-induced ROS accumulation. In this case, the effect of Doxo could be enhanced by Ferutinin action, likely due to the trigger of the biphasic elevation of intracellular calcium through the opening induction of calcium channels; the subsequent alteration of the trans-membrane potential triggers the overproduction of ROS in mitochondria at the level of the respiratory chain [62]. Furthermore, Ferutinin, through the calcium increase at pre-lethal concentrations, leads to the loss of trans-membrane potential and mitochondrial permeabilization, with the exit of molecules implicated in intrinsic apoptotic induction and progression. In addition, it has been observed that the increase in intracellular calcium induced by Ferutinin enhances NOS activity, which catalyzes NO production, which at high concentrations and for a prolonged time triggers cellular toxicity mechanisms activating several pathways of cell death and cell growth arrest [63].

Moreover, the NO bounds and inhibits the cytochrome c oxidase that leads to ROS overproduction—the consequent formation of peroxynitrite—with consequent oxidation and irreversible damage of all mitochondrial complexes, leading to apoptosis [64,65].

To confirm this hypothesis, the same concentration (2.5 μM) of FcFE, when used to pre-treat the H9c2 subsequently exposed to Doxo (1 μM and 3 μM), exacerbated its cytotoxic effects, with a higher percentage of cell death determined by a proportional inhibition of cell metabolism and deduced by the reduction in intracellular NADH-dependent dehydrogenase activity. Instead, FcFE 0.25 μM significantly reduced Doxo-induced cell death in H9c2 incubated with Doxo 0.5 μM or Doxo 1 μM, suggesting the existence of a different protective mechanism exerted by Ferutinin in counteracting the toxic effect of Doxo.

We have investigated if the anticytotoxic effect of low concentrations of Ferutinin could be due to a modulation of the cell cycle. Evidence exists that some drugs specifically attack cells in a single phase of the cell cycle [66]. Among them, Doxo is a cell cycle nonspecific agent (but is most active in the S phase) that acts by blocking topoisomerase II activity and by intercalating into the flat space between the bases of the DNA double helix, where it can act further to disrupt DNA replication and transcription [66]. The cell cycle analysis that we have conducted using the same experimental design to test cell viability showed that Doxo affects the cycle phases; in particular, there is evidence of an important reduction in the S phase. On the other hand, we observed that for FcFE 0.25 μM, when administered in pre-treatment with Doxo 1 μM—although the initial reduction of G1 phase was able to revert significantly the antiproliferative and toxic effects of the chemotherapeutic drug—restored the S phase of cell cycle and apported a significant reduction in the G2-M phase. Thus, our results suggest that FcFE, at a concentration of 0.25 μM, is able to reduce significantly the Doxo-induced mortality in rat cardiomyoblast acting on the regulation of cell cycle phases.

### Limitations

The main limitation of this study depends on the lack of the activity evaluation of FcFE at low doses on different cancer cell lines treated with Doxo, simultaneously with the studies carried out on cardiac cell line. Furthermore, the identification of underlying molecular pathways, analyzed by biomolecular assays and providing the distinction between necrosis and apoptosis, is needed. This could provide further valuable details on the mechanism of action of FcFE. These insights will be necessary before performing future in vivo toxicological and pharmacokinetic investigations.

## 4. Materials and Methods

### 4.1. Extraction Procedure and High-Pressure Liquid Chromatography (HPLC) Analysis

*Ferula communis* L. root extract was obtained from the roots of *F. communis* L. collected in Sardinia, Italy. The extract was obtained through the mixing of 25 g of root and 125 g of acetone (1:5 ratio), and the subsequent maceration, which was carried out for 60 min in the dark, as described by Maiuolo et al. [44].

Following maceration, the solution was centrifuged at +4 °C, 1036 rcf, for 5 min and filtered through filter paper. The extract was dried under vacuum through a rotary evaporator to obtain a dry powder.

High-Pressure Liquid Chromatography (HPLC) analysis was carried out on a Perkin Elmer Module (Waltham, MA, USA) equipped with a photodiode-array (PDA) detector, a series 200 autosampler, a series 200 Peltier LC column oven, a series 200 LC pump, and an Agilent 4 μm C18 100A (250 × 4.6 mm) column [67].

HPLC system control and data collection were carried out online with Chromera software (version 3.4.0.5712). The standards were purchased by Sigma-Aldrich (St. Louis, MO, USA). A total 10 mg of *Ferula* extract was dissolved in 10.0 mL of methanol. The resulting solution was vortexed for complete dissolution. The solution was filtered with a 0.2 μm PTFE filter, and 10 μL of sample were injected into the HPLC system. A two-solvent gradient (0.88% trifluoroacetic acid/acetonitrile) was used for the elution with a flow of 1 mL/min, keeping the column at 30 °C. The wavelength of the detector was set to 256 nm. Method validation parameters such as linearity, limits of detection, precision, and accuracy are available in Supplementary File S1.

The chemical composition of the powder extracted from the root of *Ferula communis* L., ssp. *glauca*, was determined by high-performance liquid chromatography (HPLC), kindly provided by the Research and Development area of Herbal & Antioxidant Derivatives (H&AD) s.r.l. (Bianco, RC, Italy). The chromatographic analysis revealed a Ferutinin concentration of 27% in the powder (Supplementary File S2).

### 4.2. Cell Culture

The study was conducted on an adherent H9c2 line of rat embryonic cardiomyocytes (ATCC, Rockville, MD, USA). The cells were cultured in Dulbecco’s modified Eagle’s medium (DMEM), with 10% fetal bovine serum (FBS), 100 U/mL penicillin, and 100 μg/mL streptomycin, into a humidified 5% CO_2_ atmosphere at 37 °C. Medium was changed every 2–3 days and cells were grown until they reached 70–80% confluency in 100 mm dishes. Cells were trypsinized and disseminated for the treatments with Ferutinin contained in *Ferula communis* L. root extract (FcFE).

*Ferula* L. extract powder was dissolved in DMSO and subsequently filtrated; then, the stock was diluted, to obtain different concentrations, in Dulbecco’s modified Eagle’s medium (DMEM) (Dmem W/Glutamax-I, Pyr,4.5g Glu-31966047-Gibco) with 10% fetal bovine serum (FBS, Tokyo, Japan) (Fbs, Qualified, Hi,10500064-Gibco), 10,000 U/mL penicillin, and 10 mg/mL streptomycin (Penicillin Streptomycin Sol, 15140122-Gibco). For determination of ROS production, we used DMEM, High Glucose, and No Phenol Red (Gibco, 21063029), which do not interfere with fluorescence evaluation. DMSO solvent used for FcFE solubilization was present in FcFE dilutions (used for treatment) at low concentrations (≤0.08%).

### 4.3. Trypan Blue Assay

The cell viability was assessed by trypan blue assay (0.4% *w*/*v*). The cell number was determined by counting the viable cells in a cell count with Neubauer chamber and the cell death rates were calculated as the percentage of stained versus total cells counted and expressed as the ratio between treated and untreated (CTRL) cells [68].

Trypan blue assay has been used to determine the effects on cell death of increasing concentration of FcFE and the potential protective effect of FcFE on Doxo-induced cell mortality. Briefly, the H9c2 cells were distributed in 6-well dishes at a density of 25 × 10^4^ and treated with different concentrations of Ferutinin contained in *Ferula communis* L. extract, corresponding to 0.15 μM, 0.25 μM, 1 μM, 2.5 μM, 5 μM, 7.5 μM, 10 μM, 12.5 μM, 15 μM, and 20 μM of Ferutinin (FcFE), and incubated for 24 h to evaluate the related viability.

In addition, to test the potential protective role of different concentrations of FcFE on cell viability, H9c2 cells were seeded in 6-well dishes at a density of 25 × 10^4^; 0.25 μM, 1 μM, and 2.5 μM of FcFE were used to pretreat cells for 3 h; subsequently, they were exposed to increasing concentrations of Doxo (0.5 μM, 1 μM, and 3 μM) for 24 h.

### 4.4. MTT Assay

Cell metabolic activity was assessed using the MTT colorimetric assay (Cell Growth Determination Kit MTT Based, CGD-1, Sigma). When taken up by metabolically active cells, MTT is converted from a yellow to a water-insoluble blue precipitate by cellular dehydrogenases [69]. Briefly, 1 × 10^4^ cells/well were plated in 96-well dishes and allowed to adhere overnight. The following day, cells were exposed to different concentrations of FcFE, corresponding to 0.15 μM, 0.25 μM, 1 μM, 2.5 μM, 5 μM, 7.5 μM, 10 μM, 12.5 μM, 15 μM, and 20 μM of Ferutinin (FcFE), and incubated for 24 h.

In addition, 1 × 10^4^ cells/well were plated in 96-well dishes to test the potential protective role of different concentrations of FcFE on metabolically active cells. In particular, 0.25 μM, 1 μM, and 2.5 μM of FcFE were used to pretreat cells for 3 h; subsequently, they were exposed to increasing concentrations of Doxo (0.5 μM, 1 μM, and 3 μM) for 24 h.

After 24 h of treatment, for both plates, 0.5 mg/mL of MTT was added to the medium with subsequent incubation at 37 °C for 4 h. The resulting blue formazan crystals were solved in DMSO. The absorbance was read on a microplate reader (Multiskan GO, Thermo 248 Scientific, Denver, CO, USA) at 570 nm and the background absorbance, measured at 690 nm, was subtracted. The absorbance of CTRL (untreated) was taken as 100% metabolically active cells.

### 4.5. Determination of ROS Production

The fluorescence assay, with 2′, 7′-dichlorodihydrofluorescein diacetate (H2DCF-DA; Molecular Probes, Eugene, OR, USA) as a probe, was used to assess ROS accumulation [70].

H2DCF-DA readily diffuses into the cells where intracellular esterases cleave the acetate group of H2DCF-DA from the molecule to yield H_2_DCF, which is trapped within the cells. Intracellular ROS [71] oxidize H_2_DCF to produce the highly fluorescent compound DCF. Briefly, H9c2 cells were sub-cultured from confluent 100 mm plates and seeded in 96-well plates. Twenty-four hours after plating, the growth medium was replaced with fresh medium containing *F. communis* L. extract at different concentrations and H_2_DCF-DA (20 mM final concentration); after incubation for 3 h at 37 °C, the fluorescence was monitored every 5 min for 60 min following the addition of H_2_O_2_ (data reported are those recorded 30 min after H_2_O_2_ addition) or after 24 h of treatment with Doxo by microplate fluorometer (Thermo Fisher Scientific, Waltham, MA, USA, excitation, 485 nm; emission, 530 nm). Data were reported as mean ± S.E.M. of eight wells per experimental group.

Increasing concentrations of FcFE were used to pre-treat H9c2 for 3 h. Subsequently, the H9c2 cells were exposed to 50 μM and 250 μM H_2_O_2_, respectively, to evaluate ROS production. Consequently, to determine the potential antioxidant effect of different concentrations of FcFE on Doxo-induced ROS production, H9c2 cells were pre-treated with 0.25 μM and 2.5 μM FcFE before the treatment with increasing concentrations (0.3 μM, 0.5 μM, and 1 μM) of Doxo. The cells were incubated for 24 h and, subsequently, the relative ROS production was assessed.

### 4.6. Cell Cycle Analysis

The *F. communis* L. root extract corresponding to an FcFE concentration of 0.25 μM was used to pre-treat the H9c2 3 h before the treatment with increasing concentrations (0.5 μM, 1 μM, and 3 μM) of Doxo. The cells were incubated for 24 h and subsequently removed by trypsinization; the adherent cells were harvested, washed twice with PBS, and fixed in 70% cold ethanol for at least 2 h at −20 °C. Fixed cells were washed with PBS, incubated with 1 mL PBS containing 0.5 mg/mL RNAse A and 0.5% Triton X-100 for 30 min at 37 °C, and stained with 50 mg/mL propidium iodide [72]. Cells were analyzed for DNA content by flow cytometry BD FACSCanto II system (BD Biosciences, Heidelberg, DE) and the percentage of cells in various cell cycle phases was determined using FACSDiva software v. 6.1.3 (BD Biosciences, Heidelberg, Germany). 

### 4.7. Statistical Analysis

The results are expressed as mean ± SEM of at least three independent experiments. The data were analyzed using GraphPad PRISM 9.3.1 (GraphPad Software, Inc., La Jolla, CA, USA). The differences among treatments were evaluated using GraphPad PRISM 9.3.1 (GraphPad Software, Inc., La Jolla, CA, USA). The Shapiro–Wilk test was using to test normality. Normally distributed data were analyzed by one-way ANOVA followed by Tukey’s test; data not normally distributed were analyzed through the Kruskal–Wallis test followed by Dunn’s tests. Comparisons of data from two groups were carried out using the Unpaired Two-tailed Student’s *t* test or Mann–Whitney test. *p*-values < 0.05 were considered statistically significant.

## 5. Conclusions

The study conducted was aimed to assess, firstly, the effect of different concentrations of FcFE on the H9c2 cell line and, consequently, the potential protective effect of low concentrations of Ferutinin when used in pre-treatment with the chemotherapeutic Doxo, which is known for its cardiotoxic effects.

This experimental activity made it possible to highlight how the *Ferula communis* L. root extract, with 27% of Ferutinin, is able to provide important cardioprotection by counteracting the effects of cardiotoxic anthracyclines, representing a useful defense in such pathological conditions also because of its antineoplastic properties. Indeed, our results suggested that FcFE could protect from Doxo-induced cardiotoxicity, opening the way for subsequent in vivo and clinical studies to evaluate the potential use of FcFE at low concentrations as an adjuvant in cancer therapy to prevent anthracycline cardiotoxicity.

Further in vitro, in vivo, and clinical trials are needed to characterize the properties of this natural extract. In fact, for the FcFE, the line between the protective and toxic effect is very tight. Indeed, the sesquiterpene Ferutinin, which represents the key component of our extract, has ambivalent properties: cellular- and dose-dependent. Therefore, the identification of the correct dosage is a fundamental step to plan this potential new therapeutic strategy. In particular, there is a need to carry out in vivo acute, subchronic, and chronic toxicity studies, followed by pharmacokinetic studies, to determine the optimal dosage for different conditions. The mechanisms of action underlying the potential beneficial effects should be investigated.

## Supplementary Materials

The following supporting information can be downloaded at: https://www.mdpi.com/article/10.3390/ijms241612735/s1.
