# Supplementary material for: In Vitro Evaluation of Ferutinin Rich-Ferula communis L., ssp. glauca, Root Extract on Doxorubicin-Induced Cardiotoxicity: Antioxidant Properties and Cell Cycle Modulation
Source: Int J Mol Sci. 2023 Aug 13;24(16):12735. doi: 10.3390/ijms241612735 (PMC10454821; doi:10.3390/ijms241612735)
Supplement: Supplementary file 1 [file ijms-24-12735-s001.zip › Supplementary S1. HPLC Methods validation parameters.pdf]

1.Linearity

10 mg of ferutinin reference standard were accurately weighted on a 20 mL volumetric flask. 10 mL of methanol were pipetted to have a stock solution of 1,000 ppm. 5 dilutions were made to have a concentration range of 1,000 – 62.5 ppm. Each solution was injected in the HPLC system in triplicate and calibration curve was made (r2=0.998).

2. Limits of Detection (LOD) and Limits of Quantitation (LOQ):

•LOD: 1 ppm

•LOQ: 3 ppm

3.Precision

Concentration (ppm)	Day 1 Peak Area	Day 2 Peak Area	Day 3 Peak Area
100	2509018	2510132	2508310
300	7512654	7506636	7512402
500	12506290	12496780	12506510
700	17500926	17489516	17499245
900	22495562	22482252	22491980

4. Accuracy:

Spiked Concentration (ppm)	Expected Concentration (ppm)	Recovered Concentration (ppm)	%Recovery
100	100	101	100.1%
300	300	298	99.3%
500	500	502	100.4%
700	700	695	99.3%
900	900	910	101.1%
