# Supplementary material for: In Vitro Evaluation of Ferutinin Rich-Ferula communis L., ssp. glauca, Root Extract on Doxorubicin-Induced Cardiotoxicity: Antioxidant Properties and Cell Cycle Modulation
Source: Int J Mol Sci. 2023 Aug 13;24(16):12735. doi: 10.3390/ijms241612735 (PMC10454821; doi:10.3390/ijms241612735)
Supplement: Supplementary file 1 [file ijms-24-12735-s001.zip › Supplementary S2. FcFE Ferula extract HPLC analysis.pdf]

SAMPLE REPORT

Acquisition Date/Time	08-Nov-21 09:52:53
Acquisition Method	Ferutinin
Chromera Version	4.2.0.6415
Dilution Factor	2175
Report Date/Time	23-May-23 11:59:21
Sample Name	Estratto ferula purificato
Vial Number	81

Estratto ferula purificato : 256:5:395:5 : 1

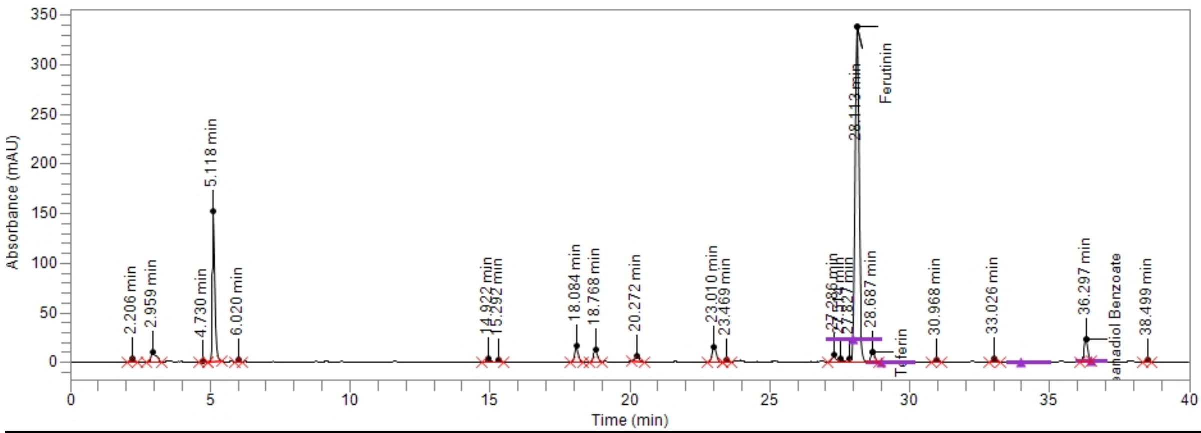

Peak #	Time	Component Name	Area	Height	Final Amount	Units
16	28.113	Ferutinin	3,130,742.0	338,602.1	270,603.9778	ppm
20	36.297	Jaeskeanadiol Benzoate	198,559.8	22,808.6		
17	28.687	Teferin	89,094.0	9,335.2		
**Total**			3,418,395.8		270,603.9778	

SAMPLE REPORT

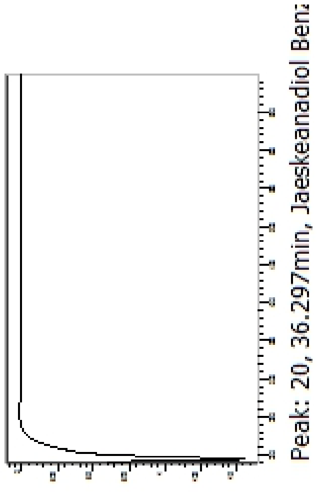

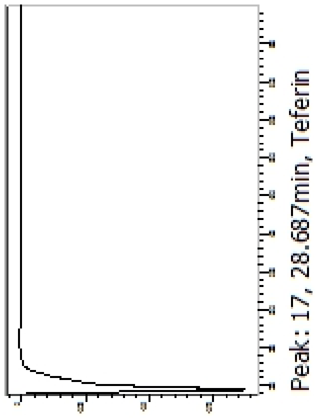

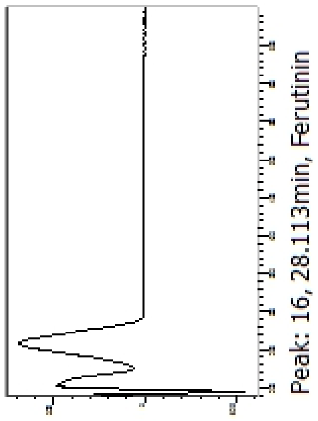

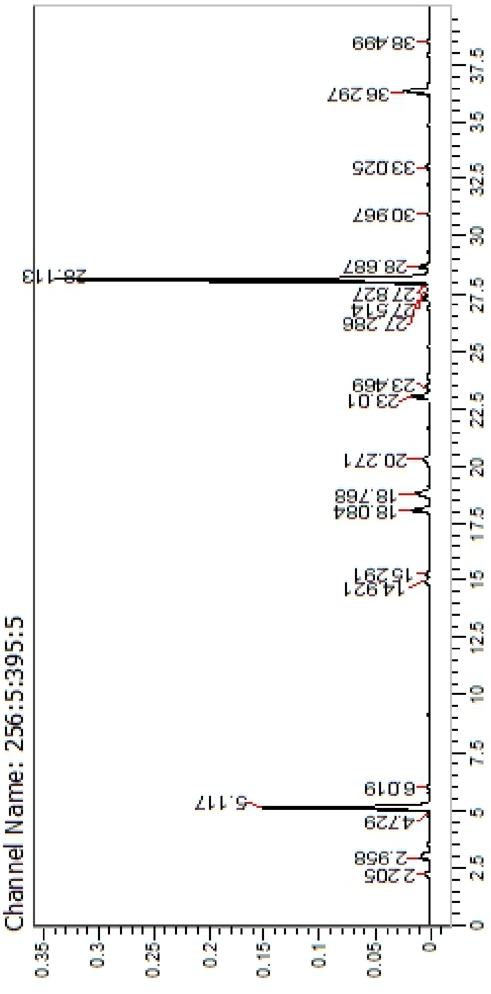

SAMPLE REPORT

Approved:

Signature: ________________ Name: ________________ Date: _____________
